# Prevalence and impact of infections in acute on chronic liver failure in Rishikesh, India: a prospective cohort study

**DOI:** 10.11604/pamj.2023.46.101.39536

**Published:** 2023-12-12

**Authors:** Beeram Krishna Prasanna Kumar, Anand Sharma, Pratima Gupta, Itish Patnaik, Rohit Gupta

**Affiliations:** 1 Department of Gastroenterology, All India Institute of Medical Sciences, Rishikesh, India

**Keywords:** Acute, chronic liver failure, sepsis, infection, drug resistance

## Abstract

**Introduction:**

infection in Acute on chronic liver failure (ACLF) is associated with poor outcomes. There is limited prospective data on microbiological and resistance profile of infections in ACLF and their impact on in-hospital mortality.

**Methods:**

the study was conducted in the Gastroenterology department of a tertiary care hospital. The study population consisted of patients hospitalized with ACLF. 123 ACLF patients were included into the study and followed till hospital discharge. Data was collected prospectively in prespecified case-record forms. The aim was to prospectively study the prevalence of bacterial infection in ACLF, compare outcomes between patients with and without infection, microbiological profile and its impact on in-hospital mortality in ACLF. Predictors of presence of infection and mortality were estimated using univariable and multivariable regression.

**Results:**

of the 123 patients included [Mean ± SD age 45.5 ± 11.8 years, Males 89%(n=110); Mean ± SD MELD: 32±8], infection was noted in 62% (n=77) patients on admission, but microbiological confirmation was present in only 35 of these who yielded 41 isolates. Spontaneous bacterial peritonitis (SBP) was the most common cause of infection, although most isolates were obtained from blood cultures. 58.5% (n=24) isolates were resistant to multiple drugs. In-hospital mortality was noted in 53% (n=65). Factors associated with in-hospital mortality on multivariable analysis were serum creatinine (aOR: 2.89, 95% CI 1.79-4.65; p < 0.01), international normalized ratio (aOR: 3.169, 95% CI 1.66-6.04; p < 0.001), infection at admission (aOR: 3.81, 95% CI 1.39-10.44, p 0.009).

**Conclusion:**

ACLF is associated with high prevalence of infection by drug-resistant organisms. Infection at admission is an independent predictor of in-hospital mortality.

## Introduction

Acute on chronic liver failure (ACLF) is a syndrome seen that occurs in cirrhosis and is characterized by systemic inflammation, organ failures and high short-term mortality. The condition represents an acute decrease in liver function and is usually associated with a precipitant and carries at least 15% 28-day mortality [1]. Cirrhosis is characterized by cirrhosis associated immune dysfunction (CAID) which consists of immune system damage and systemic inflammation [2]. Immune damage seen in cirrhosis is multifactorial. Firstly, architectural distortion due to liver fibrosis decreases bacterial clearance. Secondly, the bactericidal capacity of the innate immune system cells is reduced. Thirdly, there is increased translocation of bacteria and bacterial products across the gut due to a leaky gut, increased intestinal bacteria and intestinal dysbiosis. Finally, there is systemic immune dysfunction due to cytopenias and individual cell line dysfunction. The above changes lead to immune-exhaustion and tolerance to further increase in the infective load [2]. Systemic inflammation is due to both ligands released from necrotic hepatocytes, damage associated molecular patterns (DAMPs) and those released from bacterial products or pathogen associated molecular patterns (PAMPs) [2]. Infection is the most common precipitant of ACLF according to western literature. Massive release of PAMPs that occur in infection leads to heightened inflammation and subsequent worsening in the pre-existing hyperdynamic circulation. Consequently, there is microcirculatory disturbances leading top organ hypoperfusion and failure [3]. In addition, bacterial infection may occur as complication of ACLF. The exaggerated inflammation and immune response described above leads to immune compromise leading to increased predisposition to infection [3]. Infection related ACLF (i-ACLF) has been shown to associated with more severe disease and high 30-day mortality [4]. In a multicenter prospective study from the NACSELD database, infection associated ACLF was an independent predictor of 30-day mortality. In a study from India infection was noted in 382 out of 572 patients with acute decompensation and the authors found that it was associated with increased risk of organ failure and short-term mortality [5]. However, apart from this large study there is limited prospective data from India on impact of infections on ACLF as well as the prevalence of multidrug resistance in these patients. The primary objective of this study was the prevalence of infections in ACLF and comparison of disease severity and outcomes between patients with and without infection. The secondary objectives included the microbiological and resistance pattern of the infections as well as the factors predicting presence of infections and in-hospital mortality.

## Methods

**Study design and setting:** this was a prospective cohort study conducted on ACLF patients admitted under Department of Gastroenterology, All India Institute of Medical Sciences, Rishikesh, India. It is tertiary referral hospital.

**Study population:** consecutive patients admitted between January 2019 and August 2020 with ACLF were recruited. ACLF was diagnosed according to the European association study of liver disease (EASL) criteria [1]. Patients were excluded if they were less than 18 years of age or refused to participate in the study. Regarding sample size estimation, it was a time-based study and all consecutive patients who presented in the above duration were recruited. All patients were evaluated for presence of infection on admission to the hospital Antibiotics started empirically was Piperacillin-Tazobactam in patients who did not require inotropes and Meropenem in those requiring inotropes. Clindamycin was added in skin and soft tissue infections if necrotizing fasciitis was suspected. An antibiotic was modified according to culture reports.

**Laboratory investigations:** cultures of blood, ascitic fluid, urine, sputum (if symptoms of lower respiratory infection were present) and pus (in case of a wound) were obtained and serum Procalcitonin levels were sought before starting empirical antibiotics according to department policy.

**Data collection:** data was collected as per a prespecified case record form. Baseline characteristics, details of culture sensitivity, clinical events including in-hospital organ failure and in-hospital mortality were noted. Patients were followed till discharge from hospital.

**Definitions:** spontaneous bacteremia was defined as a positive blood culture in the absence of other sources of infection. Pneumonia was diagnosed in the presence of radiological features or sputum culture positivity. Urinary tract infection was diagnosed in patients with lower urinary tract symptoms if urine showed >10 leucocytes per high power field and/or urine culture was positive. Spontaneous bacterial peritonitis (SBP) was diagnosed if ascitic fluid neutrophil count was more than 250/cumm in the presence of positive ascitic culture. If ascitic fluid neutrophil count was >250/cumm but culture was negative patients were diagnosed as culture-negative neutrocytic ascites (CNNA). Skin and soft tissue infection (SSTI) was diagnosed in the presence of pain and redness of overlying skin with/without pus discharge and ulcer formation. Multidrug resistance was defined as resistance to at least one agent each from three categories of antibiotics. Extensively drug resistance was defined as resistance to one drug in all except two or fewer categories of antibiotics. Pan drug resistance was defined as resistance to all available agents [6]. Outcomes included organ failure and in-hospital mortality. Organ failures were defined according to the CANONIC criteria [1]. Active alcohol consumption was defined as alcohol consumption of > 30gm/day in males and >20gm/day in females in the past 60 days. MELD, Child Pugh and CLIF-C-ACLF scores were calculated as described in the original studies.

### Statistical analysis

Categorical variables were presented as number and percentages (%) and continuous variables were presented as mean ± SD or median (range) depending on the normality of data. Normality of data was tested by Shapiro-Wilk Test. Quantitative variables were compared between groups using independent t test or Mann-Whitney U test (in case of skewed data). Qualitative variables were compared using Chi-Square test / Fisher´s exact test. Comparison of normally distributed continuous variables among more than two groups were done using ANOVA test or Kruskal Wallis Test (in case of non-normal data). Parameters which were biologically plausible to affect in-hospital mortality were entered into logistic regression. As planned beforehand, initially univariable analysis was performed and those factors which were significant on univariable are entered into multivariable analysis to identify risk factors independently predicting infections and mortality. The statistical analyses were performed using the Statistical package for social sciences version 23 (SPSS, Chicago, Illinois, USA). All statistical analyses were based on two-sided hypothesis tests with a significance level of P < 0.05.

**Ethical considerations:** there were no ethical issues with respect to the study as it was an observational study. Consent was taken prior to recruitment. The study was approved by the institutional ethic committee (Letter No AIIMS/IEC/19/701).

## Results

**Baseline characteristics of the study population:** One hundred and twenty-three patients were admitted with ACLF during the study period, and all were recruited. The demographic details of the study population are detailed in Table 1. 89% of the patients were male and alcohol was the most common etiology of the acute hepatic insult and the underlying chronic liver disease. 65 patients (53%) died during hospital stay.

**Table 1:** demographic details and disease severity of 123 patients with acute on chronic liver failure on admission

Parameters	Total population (n=123)	Infection (n=77)	No Infection (n=46)	P-value
Age (Years)	45.5 ±11.8	46.9±11.1	43.2±12.7	0.09
Gender (Males)	110 (89%)	69 (56%)	41 (33%)	0.93
Prior hepatic decompensation	41 (33%)	15 (12.1%)	26 (21.9%)	0.89
Etiology of cirrhosis:				
Alcohol related	79 (65%)	49(39.8%)	30(25.2%)	
Viral	41 (31%)	22(17.8%)	19(13.2%)	
NAFLD	11(9%)	10(8.1%)	1(0.9%)	
Others	1 (1%)	1(1%)	0	
Dual etiology	9(7.3%)	5(4%)	4(3.3%)	
Number of organ failures	2.19±1.08	2.36±1.13	1.91±0.96	**0.03**
Haemoglobin (g/dL)	9.2 ± 1.9	9.37±1.83	8.9±1.9	0.2
Leucocyte count (per microliter)	15.9±9.3	16.9±9.1	14.3±9.6	0.14
Platelets (x1000/microliter)	106.9 ± 58.2	99.3±51	119.7±67.3	0.06
Creatinine (mg/dL)	2.3 ± 1.7	2.24±1.5	2.54±1.95	0.33
Total Bilirubin (mg/dL)	17.4 ± 10.5	18.6±10.3	15.5±9.6	0.11
AST (U/L)*	109 (74-190)	109 (74-188)	109(74-203)	0.86
ALT (U/L)*	52 (33-93)	52(38-95)	50(27-81)	0.35
ALP (U/L)*	149 (116-202)	146(117-199)	152(115-214)	0.77
Total Protein (g/dL)	6.2 ± 2.6	5.93±1.03	6.78±3.97	0.08
S. Albumin (g/dL)*	2.4 ± 2.84	2.41±0.42	2.39±0.47	0.81
INR	2.6 ± 1.1	2.7±1.18	2.39±0.87	0.11
MELD Score	32 ± 8	32±8	31±8	0.28
CLIF-C ACLF Score	50.4 ± 9.3	52.17±9.9	47.43±7.47	<0.001
Procalcitonin* (ng/ml)	1.9 (0.8-4.5)	2.3(0.9-6.05)	1.2(0.5-2.22)	0.007
Mortality	65 (53%)	47(38.2%)	18(24.8%)	0.02
Duration of hospital stay* (days)	9 (5-16)	9(6-16)	8(5-12)	0.07

Values are expressed as mean ± standard deviation and number (percentages). *values are expressed as median (IQR). NAFLD: Non-alcoholic fatty liver disease; AST Aspartate aminotransferase; ALT Alanine aminotransferase; ALP Alkaline phosphatase; INR: International normalized ratio; MELD: Model for end stage liver disease; CLIF-C ACLF: Chronic liver failure consortium acute on chronic liver failure

### Microbiologic profile of the patients

The sites of infection in the study patients are listed in Table 2. Infections were noted in 77 (62%) patients on admission and de novo in 18 (14.6%) patients after 48 hours of hospital stay. Cultures from 35 (45.5%) patients yielded 41 isolates on admission and 3 organisms were isolated from 3 patients after 48 hours during hospitalization. The most common type of infection identified during admission as well as hospital stay was SBP, but culture positivity was seen in only 9 patients. Bacteraemia was the most common cause of culture positivity and was noted in 17 (14%) patients on admission. Table 3 shows the organisms isolated in culture. Most cultures yielded gram-negative bacilli (92%) and Escherichia coli was the most common organism isolated. Multidrug resistance was noted in 11(26.8%) isolates and extensive drug resistance was noted in 12(29.2%) isolates. One isolate was pan drug resistant. Hence 24/41 (58.5%) isolates were resistant to multiple drugs.

**Table 2:** site of infection in patients with ACLF and bacterial infection

	Diagnosed on admission	Culture positive* on admission	Diagnosed during hospital stay	Culture positive* during hospital stay (>48 hours)
Bacteraemia	17	17	1	1
Urinary tract infection (urine culture positive)	15	5	6	1
Spontaneous bacterial peritonitis	28	8	6	1
SSTI	8	4	0	0
Pneumonia	9	1	5	0

SSTI: Skin and soft tissue infections *Culture positivity is mentioned for blood in patients with bacteraemia, urine in urinary tract infection, ascitic fluid in spontaneous bacterial peritonitis, pus in SSTI and sputum in pneumonia

**Table 3:** details of organisms isolated in cultures

	Organisms isolated on admission	Organisms isolated in hospital acquired infections
Blood culture	Escherichia coli: 6	Escherichia coli:1
	Klebsiella pneumoniae: 4	
	Stenotrophomonas: 3	
	Acinetobacter baumanii: 2	
	Staphylococcus aureus: 2	
Urine culture	Escherichia coli: 4	Klebsiella pneumoniae: 1
	Klebsiella pneumoniae: 1	
Ascitic fluid	Escherichia coli: 5	Acinetobacter baumanii: 1
	Klebsiella pneumoniae: 3	
	Staphylococcus aureus: 2	
	Enterococcus faecalis: 1	
Pus culture	Escherichia coli: 2	
	Klebsiella pneumoniae: 1	
	Acinetobacter baumanii: 1	
Sputum culture	Acinetobacter baumanii: 1	

### Comparison of patients with and without bacterial infections

Of the 123 patients with ACLF, number of patients with ACLF grade 1, 2 and 3 were 40, 39 and 44 respectively. Table 4 compares disease severity, incidence of infection and outcome between different grades of ACLF. Patients with higher grades of ACLF had significantly higher MELD and CLIF-C ACLF scores as well as higher procalcitonin levels and presence of infection. The in-hospital mortality was also more in higher grades of ACLF. Patients with infections were significantly older, had a greater number of organ failures, higher CTP score, procalcitonin levels and in-hospital mortality (Table 1). A higher proportion of NAFLD was noted in patients with infection. The average hospital stay (and hence duration of follow up) was 9(6-16) and 8(5-12) days in the infected and non-infected groups respectively (p=0.07).

**Table 4:** comparison of patients according to grade of ACLF

	ACLF-1	ACLF-2	ACLF-3	P value
Number of patients	40	39	44	
Age*	47.4±13.2	43.6±11.2	45.5±10.9	0.35
MELD score*	25±4	32±5	39±6	<0.001
CLIF-C ACLF score*	42.6 ± 5.93	48.7±5.88	58.8±7.3	<0.001
Presence of infection	22(55)	21(53.8)	34(77.3)	0.04
Procalcitonin	0.8(0.4-2.25)	1.9(0.9-3.2)	4.21.5-8.8)	<0.001
In-hospital mortality	8(20)	14(36)	43 (97.7)	<0.001

Values are expressed as mean ± standard deviation and number (percentages). *values are expressed as median (IQR). ACLF: Acute on chronic liver failure; MELD: Model for end stage liver disease; CLIF-C ACLF: Chronic liver failure consortium acute on chronic liver failure

### Factors associated with infection and in-hospital mortality

On univariable analysis serum procalcitonin and number of organ failures predicted presence of infection in ACLF and were included in multivariable analysis. However, on multivariable logistic regression, only serum procalcitonin independently predicted the presence of infection on admission (aOR= 1.143, 95% CI: 1.002-1.303, p=0.047) (Table 5). Factors associated with in-hospital mortality on univariable analysis were Bilirubin, Serum creatinine, International normalized ratio and Infection at admission. However, on multivariable analysis, infection at admission (aOR: 3.81, 95% CI 1.39-10.44, p=0.009), serum creatinine (aOR: 2.89, 95% CI 1.79-4.65; p < 0.01), International normalized ratio (aOR: 3.169, 95% CI 1.66-6.04; p < 0.001) independently predicted in-hospital mortality (Table 6).

**Table 5:** predictors of presence of infection at admission in ACLF patients

	Univariable		Multivariable	
	Unadjusted OR (95% CI)	p	Adjusted OR (95% CI)	p
Age	1.028(0.995-1.062)	0.098		
Prior decompensation	0.949(0.437-2.063)	0.895		
Hepatic encephalopathy at admission	0.55(0.242-1.25)	0.154		
Shock at admission	0.406(0.044-3.744)	0.426		
Number of Organ failures	1.491(1.032-2.155)	0.033		
Bilirubin	1.030(0.993-1.068)	0.11		
INR	1.373(0.920-2.050)	0.12		
Albumin	1.108(0.479-2.566)	0.81		
Serum Procalcitonin	1.174(1.035-1.332)	0.013	1.143(1.002-1.303)	0.047

**Table 6:** predictors of mortality in ACLF patients

	Univariable		Multivariable	
	Unadjusted OR	P value	Adjusted OR (95% CI)	P value
Bilirubin	1.05 (1.013-1.091)	0.008		
Serum creatinine	2.15 (1.5-3.07)	<0.001	2.89 (1.79-4.65)	<0.001
Serum Albumin	0.45(0.19-1.06)	0.068		
International normalized ratio	2.47 (1.47-4.13)	0.001	3.169(1.66-6.04)	<0.001
Infection at admission	2.43(1.15-5.15)	0.02	3.81(1.39-10.44)	0.009

## Discussion

This study aimed at studying prevalence of bacterial infections, their microbiological profile as well as predictors of mortality in ACLF patients. In this population of 123 patients, we reported infections in 62% patients of which 45.5% patients were culture positive. Most of the infections were caused by gram negative organisms, most of which were resistant to multiple drugs. Those with infection had higher liver disease severity scores on admission and higher in-hospital mortality. Serum Procalcitonin and number of organ failures independently predicted the presence of infection on admission. Finally, we also showed that presence of infection on admission was an independent predictor of mortality.

Infections are known to precipitate ACLF [7]. They exacerbate systemic inflammatory response syndrome (SIRS) in cirrhosis thereby precipitating ACLF and resulting in multiorgan failure and high mortality [8]. Our findings support this. Presence of infection was associated in our study with higher CLIF-C ACLF scores, organ failures and mortality. In the CANONIC study, bacterial infections were present in 32.6% of patients with ACLF, whereas the group without ACLF showed significantly lower prevalence of infection [1]. Other studies in ACLF patients have shown the prevalence of bacterial infections to be between 27-41% [3,9,10]. While most of the studies reported SBP to be the most common site of infection [1,3,9], urinary tract infections (UTI) were the most common source of infection in the NACSELD study followed by SBP [10]. In our study, the prevalence of infections was much higher at 62%, though microbiological confirmation was observed in only 45.5% of the patients. Our findings are concordant with a previous study from India which reported infections in 66.8% of patients [5]. However, pneumonia was the most common source of infection in the above cohort [5], while most of our study patients had SBP. Absence of culture positivity in more than half of the patients with infection may be associated with the fact that most of our patients receive initial medical are from community practitioners and are likely to have received antibiotics prior to presenting to us.

In our study, serum procalcitonin was the only independent predictor of infection at admission. Procalcitonin levels were significantly greater in the group with infection than the one without. However, notably, even the group without infection had procalcitonin levels higher than 0.5ng/ml that is commonly used as a cut-off for diagnosing infection in non-cirrhotic patients. This is concordant with previous studies which have reported high procalcitonin levels in advanced liver disease irrespective of infection [11,12]. This is thought to be due to endotoxemia, damage associated molecular patterns (DAMPs) and induction of procalcitonin synthesis by TNF-α and IL-1β [13].

Bacterial infection has been associated with increased disease severity and mortality in multiple other studies. In a longitudinal study of 407 patients with ACLF, those with infection at diagnosis exhibited higher systemic inflammation and mortality. Bacterial infection was an independent predictor of mortality in this study [3]. Another study included 173 patients with ACLF and identified infection triggered ACLF to be independently associated with mortality [9]. Similar findings have also been reported from India [5]. In our study, presence of infection predicted mortality independently after adjusting for the three components of MELD score, viz. Bilirubin, INR and creatinine as well as albumin, which assesses nutritional status. Another notable finding in our study is that 24 out of 41(58.5%) isolates were resistant to multiple antibiotics. Patients with cirrhosis undergo multiple hospitalizations, invasive interventions and frequently receive prophylactic and therapeutic antibiotics, thereby predisposing them to develop MDR infections. Fernández *et al*. reported prevalence of MDR infections in 15.4% of 455 patients with infections [6]. However, in an Italian study 51.5% of the isolates in patients with cirrhosis showed resistance to multiple antibiotics, thereby causing failure of empirical antibiotics [14]. In another study from India, the proportion of MDR infections were 29% [5]. Our patients mostly come from mountainous regions with poor healthcare facilities. The higher proportion of drug resistance in these patients may be related to the lack of awareness of antibiotic stewardship practices in remote areas.

Our study findings imply that infection should be aggressively looked for in ACLF since it is associated with poor outcomes. However, the yield of bacterial culture rates may be suboptimal and hence further research should be aimed at identifying novel methods of detection of bacterial growth. Another implication of our study findings is that multidrug resistance should be strongly suspected in these patients. However, our study has few limitations. Firstly, most of our patients presented first to community practitioners and were likely to have received antibiotics prior to coming to our centre. The administration of antibiotics prior to culture may have reduced the culture positivity in some patients. Secondly, our follow up was only till hospital discharge. Longer follow up is likely to provide better insight on the association of infection at baseline with outcomes as well as recurrence of infection during the follow up period.

## Conclusion

Our study demonstrates that infections at admission in hospitalized ACLF patients is associated with more severe disease and higher grades of ACLF. Serum Procalcitonin predicts infection in ACLF patients. Hence, all ACLF patients should undergo rigorous screening for infections, which should include serum procalcitonin in addition to appropriate cultures. Multidrug resistance should be strongly suspected in these patients.

### What is known about this topic

- *Cirrhosis is associated with cirrhosis associated immune dysfunction which causes increased susceptibility to infections;*
- *Infection can precipitate ACLF in patients with cirrhosis;*
- *Infection associated ACLF is associated with end organ failure*.

### What this study adds

- *ACLF is associated with high prevalence of drug resistance;*
- *Procalcitonin is an independent predictor of infection;*
- *Infection in ACLF is an independent predictor of in-hospital mortality*.
